# Identification of consensus biomarkers for predicting non-genotoxic hepatocarcinogens

**DOI:** 10.1038/srep41176

**Published:** 2017-01-24

**Authors:** Shan-Han Huang, Chun-Wei Tung

**Affiliations:** 1 Ph.D. Program in Toxicology, Kaohsiung Medical University, Kaohsiung, Taiwan; 2 School of Pharmacy, College of Pharmacy, Kaohsiung Medical University, Kaohsiung, Taiwan; 3 Research Center for Environmental Medicine, Kaohsiung Medical University, Kaohsiung, Taiwan; 4 National Institute of Environmental Health Sciences, National Health Research Institutes, Miaoli County, Taiwan

## Abstract

The assessment of non-genotoxic hepatocarcinogens (NGHCs) is currently relying on two-year rodent bioassays. Toxicogenomics biomarkers provide a potential alternative method for the prioritization of NGHCs that could be useful for risk assessment. However, previous studies using inconsistently classified chemicals as the training set and a single microarray dataset concluded no consensus biomarkers. In this study, 4 consensus biomarkers of A2m, Ca3, Cxcl1, and Cyp8b1 were identified from four large-scale microarray datasets of the one-day single maximum tolerated dose and a large set of chemicals without inconsistent classifications. Machine learning techniques were subsequently applied to develop prediction models for NGHCs. The final bagging decision tree models were constructed with an average AUC performance of 0.803 for an independent test. A set of 16 chemicals with controversial classifications were reclassified according to the consensus biomarkers. The developed prediction models and identified consensus biomarkers are expected to be potential alternative methods for prioritization of NGHCs for further experimental validation.

Carcinogenicity is one of the most unwanted side effects during drug development^1^. Carcinogenic agents can be classified as genotoxic or nongenotoxic carcinogens according to their mechanism of action. While genotoxic carcinogens directly interact with DNA, nongenotoxic carcinogens induce tumors in indirect manners^2^. As liver is the major target organ of drug-induced tumor formation, hepatocarcinogenicity has been extensively studied for decades. In contrast to genotoxic agents that can be easily identified using well-established bioassays^3^, the identification of nongenotoxic hepatocarcinogens (NGHCs) still relies on 2-year rodent bioassays^1^,^4^. The development of efficient methods is desirable for identifying NGHCs.

Toxicogenomics (TGx) as a promising tool in risk assessment^5^ has been applied to decipher the mechanism of hepatocarcinogenicity through the identification of gene biomarkers from short-term microarray experiments. To achieve reproducibility and comparability using similar study designs and standardized experimental protocols^6^, several large-scale microarray experiments were conducted to support TGx studies that are publicly available as DrugMatrix^7^, GSE8858^8^, and TG-GATEs^1^. The three datasets were comprised of time-series measurements of gene expressions at single or multiple dose levels, which were profiled using Affymetrix (DrugMatrix and TG-GATEs) and Codelink (DrugMatrix and GSE8858) platforms. Prediction models were subsequently developed based on the biomarkers for detecting NGHCs^1^,^4^,^7^,^8^,^9^,^10^,^11^,^12^. The superior performance of TGx over quantitative structure-activity relationship models for hepatocarcinogenicity has been reported^8^. However, the lack of consensus biomarkers identified from different studies could limit the usefulness for predicting NGHCs.

The utilization of different NGHCs as training sets, algorithms for biomarker identification and experimental parameters such as the duration of treatment and microarray platforms could result in the identification of different biomarkers. For example, Fielden *et al*. applied statistical methods to identify 23 biomarkers for predicting NGHCs based on a training set of 23 NGHCs and 49 NHCs and 5-day gene expression data from DrugMatrix^7^. Uehara *et al*. utilized support vector machine algorithms to conclude 9 biomarkers based on a training set of 6 NGHCs and 54 NHCs and 28-day gene expression data from TG-GATEs^1^. They further reported an improved model based on 42 biomarkers and a larger dataset of 41 NGHCs and 52 NHCs and 28-day gene expression data^13^. Masayuki *et al*. identified 106 biomarkers from six NGHC compounds based on 28-day gene expression data from TG-GATEs^14^. Eichner *et al*. present two novel approaches employed to capture 5 specific biomarkers both statistical and machine learning-based methods based on a training set of 2 Genotoxic hepatocarcinogens, 9 NGHCs and 11 NHCs and 14-day gene expression data from TG-GATEs^15^. The utilization of microarray data from different laboratories or platforms would result in a natural question whether the biomarkers are comparable and reliable^16^. The identification of consensus biomarkers could largely help the characterization and prediction of hepatocarcinogens.

This study proposed to identify and analyze consensus biomarkers based on a largest set of 50 NGHCs and 224 non-hepatocarcinogens (NHCs) without controversial classification of NGHC or NHC collected from literatures^1^,^4^,^7^,^8^,^9^,^10^,^12^,^17^,^18^,^19^, and corresponding 1-day gene expression data from 4 publicly available microarray experiments. Four differentially expressed genes of A2m, Ca3, Cxcl1 and Cyp8b1 were identified as consensus biomarkers. Machine learning models were subsequently developed based on the consensus biomarkers to evaluate the prediction performance for NGHCs and reanalyze 16 inconsistently classified chemicals. The four biomarkers achieve good performance based on a bagging algorithm with area under the receiver operating characteristic curve (AUC) values of 0.775, 0.793, 0.717 and 0.740 for four microarray datasets. The prediction model provides a potentially useful method for identifying NGHCs.

## Materials and Methods

### Chemical list

To collect a largest set of NGHCs and NHCs for the identification of consensus biomarkers for identifying NGHCs, chemicals with classification information were collected from several studies associated with nongenotoxic hepatocarcinogenicity^1^,^4^,^7^,^8^,^9^,^10^,^12^,^17^,^18^,^19^. Hepatocarcinogens with both genotoxic and non-genotoxic mechanisms were classified as NGHCs, while hepatocarcinogens with only genotoxic mechanisms were excluded. A total of 274 chemicals (50 NGHCs and 224 NHCs) without inconsistent classifications were utilized as the final chemical list for biomarker identification. There are 16 inconsistently classified chemicals with both NGHC and NHC classifications in different studies. The final chemical list is available in Supplementary Table S1.

### Microarray datasets

Four large-scale gene expression profiles and metadata named DMA, DMC, GSE8858, and TG-GATEs were downloaded from websites of DrugMatrix (ftp://anonftp.niehs.nih.gov/drugmatrix/), GSE8858 (ftp://ftp.ncbi.nlm.nih.gov/geo/series/GSE8nnn/GSE8858/) and TG-GATEs (ftp://ftp.dbcls.jp/archive/open-tggates/) as shown in Table 1. All of the four datasets contain gene expression profiles from liver samples of Sprague-Dawley rats treated with various chemicals. All the gene expression values were log2-transformed for subsequent analysis.

### Identification of consensus biomarkers

To identify consensus biomarkers from multiple microarray datasets, differentially expressed genes (DEGs) were firstly analyzed for each of the four microarray datasets. The *t*-test (*p* < 0.05) and 1.5-fold change were applied to identify DEGs based on original expression values. The 1-day gene expression profiles from the treatment of chemicals using the single maximum tolerated dose or high dose were utilized for analysis due to their availability on all four datasets. Subsequently, the overlapped DEGs from four datasets were identified as consensus biomarkers.

### Machine learning models and cross-validation

Machine learning methods have been widely used for constructing classifiers/hypothesis that can explain complex relationships in the data^20^. In this study, a total of 6 machine learning algorithms were applied to evaluate the prediction performance of biomarkers including decision tree (J48), boosting tree, bagging tree, RandomForest, Naïve Bayes and K-nearest neighbors (*k*NN). The implementation of the machine learning algorithms was based on WEKA machine learning package^21^. A brief introduction to the 6 machine learning algorithms was summarized as follows.

The decision tree algorithm J48, also known as C4.5 algorithm^22^, is a tree-based classifier that has been extensively applied in related bioinformatics problems such the identification of biomarkers^23^ and the prediction of NGHC^24^,^25^,^26^. In this study, the default confidence parameters of the confidence threshold and minimal number of samples in the left node were utilized for tree pruning to avoid overfitting.

In addition to the single decision tree classifier, three ensemble learning algorithms of boosting, bagging and RandomForest were implemented to leverage the power of multiple classifiers that have been shown to improve the overall prediction accuracy and reduce overfitting problems^27^,^28^. The boosted decision tree classifier was developed by applying AdaBoost.M1 algorithm^29^ to create an accurate classifier by combining many relatively weak decision trees. The weak classifiers were sequentially built with modified weights of samples. For each iteration, the samples correctly classified by many of the previous weak classifiers get a lower weight, and the misclassified samples get a higher weight. The AdaBoost.M1 algorithm hereby focuses on the samples with higher weights from the previous classifier. The number of trees is a parameter that should be tuned to achieve the highest performance. In this study, the area under the receiver operating characteristic curve (AUC) value was utilized for parameter tuning of the number of trees (ranged from 5 to 50). The final prediction is based on a majority vote rule from all weak classifiers.

The bagging algorithm based on the combination of bootstrap sampling and aggregation methods has been shown to effectively reduce variance and avoid overfitting^30^. For creating a bagging decision tree classifier, multiple decision tree classifiers were trained on individual random subsets that are bootstrap sampled from the training dataset. The prediction is made by aggregating prediction results from the constructed classifiers using a majority vote rule. In this study, the boosting and bagging algorithms were both implemented using the decision tree algorithm J48 as their base learner. AUC values were utilized for determining the optimal number of trees (ranged from 5 to 50).

RandomForest^31^ is another popular decision tree-based ensemble algorithm that has been shown to be useful in many applications^32^,^33^,^34^,^35^. The RandomForest classifier aims to reduce variance to avoid overfitting problems and improve prediction performances of decision trees by training a set of fully grown decision trees utilizing bootstrap samples from the training dataset and randomly selected features^36^,^37^,^38^. The number of features for constructing a fully grown decision tree is set to 1 + log2(the number of features). The optimal number of trees ranged from 5 to 50 was determined based on AUC performance. The prediction of a given sample is based on a majority vote.

In addition to the decision tree and ensemble methods, two classifiers were also evaluated for their prediction performance of the selected markers including Naïve Bayes and K-nearest neighbor (*k*NN) classifiers. Naïve Bayes method is a simple yet powerful classification method based on the Bayes’ theorem^39^. Given a hypothesis *H* and an evidence *E*, the posterior probability can be calculated based on a prior probability and a likelihood using the following equation: 
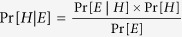
. Although it is based on a naïve assumption that features are independent, many studies showed its excellent performance for various applications^40^,^41^,^42^.

The instance-based learning algorithm *k*NN is a non-parametric classification method. The new sample was classified according to the classes of *k* nearest training samples by a majority vote rule. Euclidean distance was used to calculate the distance between two samples to identify the nearest training samples. The optimal number of nearest neighbors *k* ∈ {1, 3, 5} for KNN was determined using the AUC performance.

The development of machine learning classifiers and selection of optimal parameters were only based on the training dataset. To avoid overfitting in the training process, a leave-one-out cross-validation (LOOCV) method was utilized to evaluate the performance of selected markers on the training dataset. For each iteration, a sample is selected to validate the model constructed by using the remaining samples. After all samples are evaluated, the LOOCV performance is obtained by calculating the overall AUC performance.

### Independent test

Johnson and Johnson (JNJ) dataset^9^ was used for independent test of the developed models. The JNJ dataset consisting of 1-day gene expression data for 10,399 probes was obtained from Codelink platform based on the treatment of 9 NGHCs and 54 NHCs on Sprague-Dawley rats using single maximum tolerance doses. The JNJ dataset was download from the public database of Chemical Effects in Biological Systems (CEBS) (ftp://157.98.192.110/ntp-cebs/datatype/microarray/J&J/) and log2-transformed.

## Results and Discussion

### Inconsistency among previously identified biomarkers for NGHC

**Figure 1:**
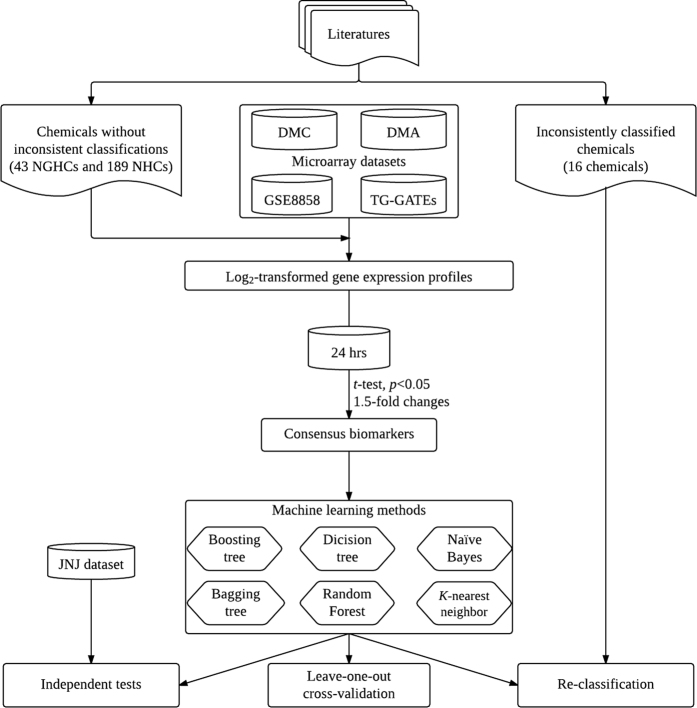
Flowchart of consensus biomarker identification and independent test. A chemical list was firstly collected from literature. Secondly, consensus biomarkers were identified based on the chemical list without inconsistent classifications. Prediction models based on machine learning algorithms were subsequently constructed using the consensus biomarkers and validated on an independent test JNJ dataset. Finally, inconsistently classified chemicals were reanalyzed using the developed models.

Previous studies reported several sets of NGHC biomarkers based on only a single dataset with different chemical lists, experimental designs, and microarray platforms^1^,^4^,^7^,^8^,^9^,^10^,^12^,^17^,^18^,^19^. To give an overview, the overlaps between published biomarkers have been collected and analyzed. As shown in Supplementary Figure S1, there is no common biomarker among the previous studies^1^,^7^,^8^,^13^,^14^,^24^. A consensus set of biomarkers is desirable for predicting NGHCs and providing better insights into the underlying mechanisms. Figure 1 shows the system flow of this study for the identification of consensus biomarkers.

### Construction of a chemical list for the identification of biomarkers

This study aimed to identify consensus biomarkers based on multiple gene expression datasets of a relatively large chemical list without inconsistent classifications from previous studies for predicting NGHCs. A large chemical list was firstly collected from literatures consisting of 50 NGHCs and 224 NHCs (Supplementary Table S1). Subsequently, gene expression data matching the 274 chemicals were extracted for identifying differentially expressed genes. After removing chemicals without corresponding gene expression data from DMA, DMC, GSE8858 and TG-GATEs datasets, a total of 232 chemicals (43 NGHCs and 189 NHCs) were utilized for the identification of consensus biomarkers.

### Identification of consensus biomarkers

Among the four microarray datasets, the common exposure styles are 1-day single dose and 3-day repeated doses using a highest tolerated dose. This study focused on the data of 1-day single dose because of its potential applications. To identify consensus biomarkers, only gene expression profiles derived from the exposure style were utilized. For each of the four microarray datasets, differentially expressed genes (DEGs) were firstly identified using t-test (*p* < 0.05) and 1.5-fold changes on original expression values between NGHCs and NHCs. The numbers of DEGs are 142, 155, 231 and 1280 for DMA, DMC, GSE8858 and TG-GATEs, respectively. Finally, 4 common DEGs of A2m, Ca3, Cxcl1 and Cyp8b1 in all four microarray datasets were identified as consensus biomarkers. A Venn diagram showing the overlap between datasets was generated by jvenn^43^ and shown in Supplementary Figure S2.

The means of log2-transformed expression values of the 4 consensus biomarkers in four microarray datasets for NGHCs and NHCs were shown in Supplementary Table S2. As expected, platform variation existed that the expression values were similar on the same platforms (DMC and GSE8858 from Codelink platform; DMA and TG-GATEs from Affymatrix) but different from other platforms. Different dosages of chemicals could also affect the expression values to vary between different microarray datasets. Details on dosages for the four microarray datasets were available in Supplementary Table S3. Generally, down-regulated expression of the four biomarkers could be the predictive markers for NGHC.

### Development of prediction models using consensus biomarkers

**Figure 2:**
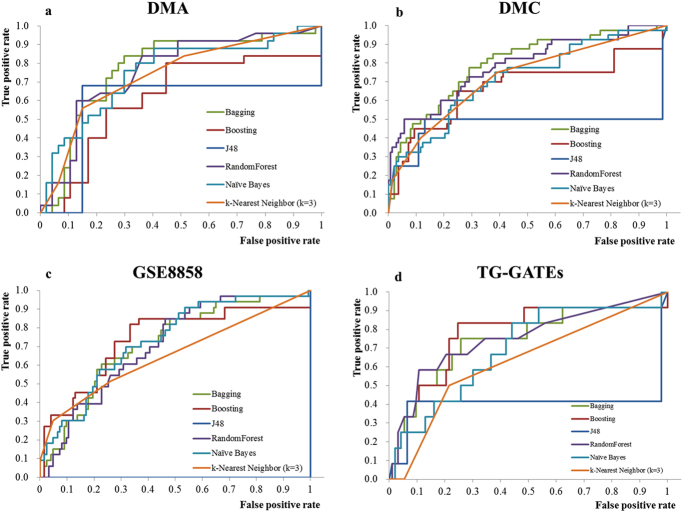
ROC curves representing the LOOCV performance of consensus biomarkers on four microarray datasets.

To evaluate the prediction ability of the four consensus biomarkers, prediction models were firstly developed by applying machine learning techniques whose prediction performance were evaluated based on leave-one-out cross-validation (LOOCV). Due to the platform variations are shown in Supplementary Table S2, four prediction models were separately constructed for each of the four microarray datasets. A total of six machine learning algorithms including J48, boosted decision trees, bagging decision trees, RandomForest, *k*NN and Naïve Bayes classifier were evaluated for developing best prediction models giving highest AUC performances. Please note that the support vector machines without a built-in mechanism for dealing missing data were not assessed in this study. Figure 2 shows the LOOCV performance of the consensus biomarkers using various algorithms for datasets of DMA, DMC, GSE8858 and TG-GATEs. For *k*-nearest neighbor classifier, the best parameter of *k* giving the highest AUC performance is 3. Among the six algorithms, bagging decision trees perform consistently well with AUC values of 0.775, 0.793, 0.717 and 0.740 using 15, 15, 48 and 48 trees for DMA, DMC, GSE8858, and TG-GATEs, respectively.

**Figure 3:**
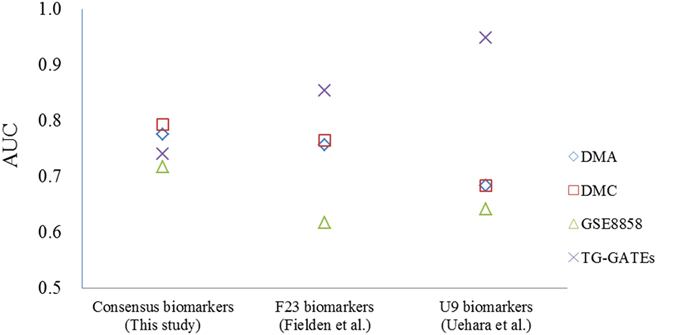
LOOCV performances for consensus biomarkers and published biomarkers.

To compare with previously published biomarkers, bagging decision tree algorithm were also applied to evaluate their LOOCV performance. Two biomarker sets consisting of 23^7^ and 9 genes^1^ named F23 and U9, respectively, were utilized for performance comparison. As the Codelink platform consisted of fewer probes, only 10 out of 23 the probes from F23 and 2 out of 9 probes from U9 were utilized for evaluating prediction performance for datasets of DMC and GSE8858. As shown in Fig. 3, the consensus biomarkers from this study achieved consistent prediction performance. The other two biomarker sets identified from previous studies performed worse than the consensus biomarkers in three microarray datasets expect for TG-GATEs. The high performance of the other two biomarker sets for TG-GATEs might be overestimated because 10 out of 12 NGHCs in TG-GATEs were associated with oxidation stress-related mechanisms and the two biomarker sets were selected based on the mechanism. In general, the average AUC value of 0.803 using the consensus biomarkers is higher than 0.572 and 0.602 using previously published biomarker sets.

Recently, Li *et al*. has proposed a resampling statistics for evaluating the robustness of biomarkers^44^. To address the robustness issue, a similar approach has been adopted for assessing the consensus biomarkers. First, a total of 60 resampling sub-datasets were constructed by randomly selecting 80 chemicals from the DMC dataset. Each sub-dataset maintained the same ratio of NGHCs and NHCs as that of the original set, with <50% sample overlap among the sub-datasets. Subsequently, the 60 distinct random sub-datasets were utilized for assessing the robustness of the 4 consensus biomarkers and 4 biomarker subsets, each consisting of 3 out of the 4 consensus biomarkers. Based on the bagging algorithm, the 4 consensus biomarkers generate an averaged AUC value of 0.701 ± 0.081 (mean ± SD), while the biomarker subsets generate a slightly lower AUC value of 0.679 ± 0.075. A certain degree of variation was observed. The robustness issue might be further studied by applying a knowledge-based CSS (combinatory cancer hallmark–based gene signature sets) approach^45^. The CSS approach based on a resampling-based Multiple Survival Screening (MSS) algorithm is capable of selecting a robust set of biomarkers from a set of mechanism-based candidate genes and might be further applied for identifying biomarkers of NGHCs.

### Independent test of consensus biomarkers

**Figure 4:**
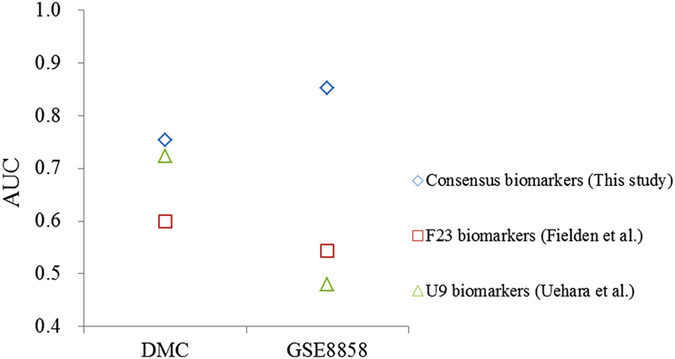
Independent test performances for consensus biomarkers and published biomarkers.

To further evaluate the prediction ability of the identified consensus biomarkers, a JNJ dataset consisting of 1-day single maximum dose exposures of 9 NGHCs and 45 NHCs^9^ was utilized to independently test the developed models. Figure 4 showed the prediction performances using the consensus biomarkers and two published biomarker sets. Since the JNJ dataset is based on Codelink platform, only models trained on DMC and GSE8858 datasets were applied to independently predict chemicals from the JNJ dataset. The consensus biomarkers achieved highest performances with AUC values of 0.753 and 0.852 for DMC and GSE8858, respectively. The other two biomarker sets performed much worse with AUC values of 0.6 and 0.544 for DMC and GSE8858 using 10 biomarkers from F23, and of 0.723 and 0.48 for DMC and GSE8858 using 2 biomarkers from U9, respectively. A poor performance (AUC = 0.49) on the JNJ dataset using F23 biomarkers was also shown in the previous study^7^. A possible reason for incorrect classification of the JNJ dataset might be the different exposure styles. Their model was developed using biomarkers identified from a microarray dataset of 5-day repeated doses, while the test is performed on the JNJ dataset of 1-day single dose^7^. The model constructed in this study using the same exposure style (1-day single dose) largely improved the performance of F23 biomarkers with an AUC of 0.6. Altogether, our consensus biomarkers worked fine for all tested datasets that were expected to be useful in future applications while the others were specific for a given dataset. The results also suggested that datasets from consistent exposure styles were more predictable. This study utilized datasets of coherent exposure styles of 1-day single highest tolerated dose achieve good prediction performance.

### Analysis of the consensus biomarkers

There are four consensus biomarkers identified in this study including Ca3, Cyp8b1, A2m, and Cxcl1. The possible roles of the biomarkers for NGHC were discussed as follows. Carbonic anhydrase III (Ca3) is a cytosolic protein found in skeletal muscle, liver, and adipose tissue of mammals^46^. A significant decrease in Ca3 expression levels was found in rat hepatocarcinogenesis^47^. The downregulation of Ca3 levels by the treatment of ethanol and a hepatotoxicant of carbon tetrachloride was also reported^12^,^48^,^49^. The depletion of Ca3 is also a useful biomarker for liver injury^50^. The usefulness of downregulated Ca3 levels for identifying NGHCs has also been demonstrated in this study.

Cyp8b1 (cytochrome p450, family 8, subfamily b, polypeptide 1) is a key enzyme involved in bile acid biosynthetic pathway which converts chenodeoxycholic acid to cholic acid^51^. An animal study showed the suppression of Cyp8b1 mRNA levels in tumorigenic hepatitis^52^. This could result in shunting toward alternative pathways, oxidative damage, and activation of pro-inflammatory second messengers, as well as disrupted signaling through lipid-sensitive nuclear receptors, such as peroxisome proliferator–activated receptors and retinoid X and liver X receptors^53^.

A2m and Cxcl1 are two novel biomarkers identified in this study that have not been directly associated with non-genotoxic hepatocarcinogenesis. A2m (a_2_-macroglobulin) is a member of the thiol ester protein family whose mRNA levels in rat liver were found to be linked to acute inflammation^54^ and NGHC-induced tumorigenesis^55^,^56^,^57^. Cxcl1 (C-X-C motif chemokine ligand 1) was found to be associated with drug-induced liver injury^58^, cirrhosis^59^, and hepatitis^60^. The two biomarkers could be further investigated to give insights into the mechanism of non-genotoxic hepatocarcinogenesis.

### Reclassification of inconsistently classified chemicals

**Figure 5:**
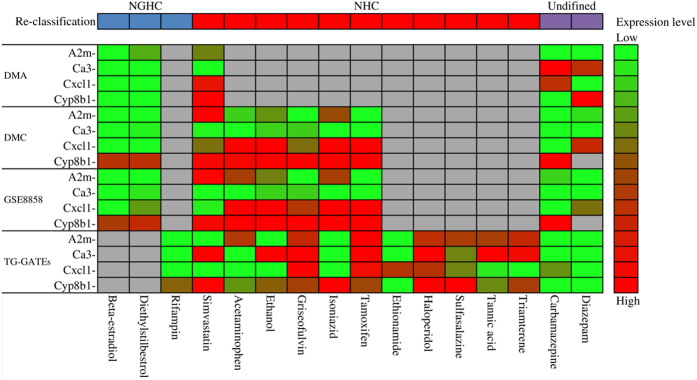
Heatmap representing the expression levels of consensus biomarkers for reclassifying chemicals based on four microarray datasets. Grey color indicates no available data.

The classification of several NGHCs is still controversial due to different criteria and doses and could change according to new evidence. During the construction of chemical lists, a total of 16 chemicals were annotated with inconsistent classifications with both NGHC and NHC from literature. A summary of the 16 inconsistently classified chemicals and corresponding maximum tolerated doses is shown in Table 2. The analysis of the inconsistently classified chemicals based on the consensus biomarkers could provide mechanism-based reclassification. The developed bagging decision tree models were applied to reanalyze the 16 chemicals based on the consensus biomarkers. Figure 5 represented a heatmap showing the expression levels of the consensus biomarkers on four microarray datasets and reclassification for the 16 chemicals. Three out of the 16 chemicals of beta-estradiol, diethylstilbestrol and rifampin were consistently classified as NGHCs. Among the 11 chemicals reclassified as NHCs, 5 chemicals were analyzed solely by the model built on TG-GATEs due to the availability of expression data including ethionamide, haloperidol, sulfasalazine, tannic acid and triamterene. Chemicals of acetaminophen, ethanol, griseofulvin, isoniazid and tamoxifen were reclassified as NHCs based on gene expression profiles from DMC, GSE8858, and TG-GATEs. Simvastatin was classified as NHC based on a majority rule that models of DMA, DMC and GSE8858 support the classification except for the TG-GATEs model. The difference of simvastatin doses might lead to inconsistent classification (1200 mg for DMA, DMC, and GSE8858; 400 mg for TG-GATEs). The remaining two chemicals of carbamazepine and diazepam were still undefined due to conflict results from the models. Carbamazepine was classified as both an NGHC (DMC and GSE8858 models) and NHC (DMA and TG-GATEs models). Diazepam was classified as an NGHC (DMA and DMC models) and NHC (GSE8858 and TG-GATEs models).

## Conclusion

The identification of consensus biomarkers based on multiple microarray datasets could provide better insights into the mechanism of NGHCs. This study identified four consensus biomarkers based on a large collection of chemical lists and four microarray datasets for predicting NGHCs and reanalyzed chemicals with inconsistent classification. Bagging decision tree models were subsequently developed for predicting NGHCs with average AUC values of 0.756 and 0.803 for LOOCV and independent test, respectively. The inconsistently classified chemicals were reclassified according to the consensus biomarkers. The developed models and biomarkers could be useful for prioritizing NGHCs for further experimental validation that could be a potential alternative to the 2-years rodent bioassay.

## Additional Information

**How to cite this article:** Huang, S.-H. and Tung, C.-W. Identification of consensus biomarkers for predicting non-genotoxic hepatocarcinogens. *Sci. Rep.* **7**, 41176; doi: 10.1038/srep41176 (2017).

**Publisher's note:** Springer Nature remains neutral with regard to jurisdictional claims in published maps and institutional affiliations.

## Supplementary Material

Supplementary Information

Supplementary Information

Supplementary Information

Supplementary Information

## Figures and Tables

**Table 1:** Summary of microarray datasets.

Dataset	#Probe	#NGHC	#NHC	Description	Reference
DMAAffymetrix whole genome 230 2.0 rat GeneChip® array	31099	25	47	■ National Toxicology Programs of the National Institute of Environmental Health Sciences in U.S.A■ 3 doses: Low, Medium and High■ 4 time points: 0.25, 1, 3 and 5 days	Fielden *et al*.^7^
DMCThe GE Codelink™ rat array	10399	39	138		
GSE8858CodeLink UniSet Rat I Bioarray	10399	35	121	■ Maximum tolerated dose■ 3 time points: 1, 3 and 5 days	Liu *et al*.^8^
TG-GATEsAffymetrix Rat Genome 230 2.0 array	31099	12	93	■ The TGx Project in Japan■ 3 doses: Low, Medium, High■ 8 time points: 3, 6, 9 and 24 hours; 3, 7, 14 and 28 days	Uehara *et al*.^1^

**Table 2:** The dosage and classification of inconsistently classified chemicals.

Chemical	Dosage (mg/kg)				Classification	
	DMA	DMC	GSE8858	TG-GATEs	NGHC	NHC
Acetaminophen		972	972	1000	Fielden *et al*.^7^	Uehara *et al*.^10^
Beta-estradiol	150	150	150		Fielden *et al*.^7^	Liu *et al*.^8^
Carbamazepine	490	490	490	300	Fielden *et al*.^7^	Uehara *et al*.^10^
Diazepam	710	710	710	250	Yamada *et al*.^12^	Fielden *et al*.^4^
Diethylstilbestrol	2.8	280	280		Fielden *et al*.^7^	Liu *et al*.^8^
Ethanol		6000	6000	4000	Fielden *et al*.^7^	Uehara *et al*.^10^
Ethionamide				250	Yamada *et al*.^12^	Uehara *et al*.^10^
Griseofulvin		2500	2500	1000	Yamada *et al*.^12^Liu *et al*.^8^	Uehara *et al*.^10^
Haloperidol				30	Yamada *et al*.^12^	Uehara *et al*.^10^
Isoniazid		79	79	2000	Liu *et al*.^8^	Uehara *et al*.^10^
Rifampin				200	Uehara *et al*.^10^	Yamada *et al*.^12^
Simvastatin	1200	1200	1200	400	Fielden *et al*.^7^	Uehara *et al*.^10^
Sulfasalazine				1000	Yamada *et al*.^12^	Uehara *et al*.^10^
Tamoxifen		64	64	60	Yamada *et al*.^12^	Uehara *et al*.^10^
Tannic acid				1000	Yamada *et al*.^12^	Uehara *et al*.^10^
Triamterene				150	Yamada *et al*.^12^	Uehara *et al*.^10^
