# Supplementary material for: Identification of consensus biomarkers for predicting non-genotoxic hepatocarcinogens
Source: Sci Rep. 2017 Jan 24;7:41176. doi: 10.1038/srep41176 (PMC5259716; doi:10.1038/srep41176)
Supplement: Supplementary Information [file srep41176-s1.pdf]

Supplementary Information files

Supplementary Figure S1. The number of common biomarkers of published studies

	Fielden et al. 2007	Liu et al. 2011	Uehara et al. 2011	Fielden et al. 2011	Eichner et al. 2014	Tung & Cheng 2014	Yamada et al. 2015	Masayuki et al. 2016
Fielden et al 2007	22							
Fielden et al. 2011	7	90						
Uehara et al. 2011	1	0	82					
Liu et al. 2011	1	0	2	35				
Eichner et al. 2014	0	0	3	2	45			
Tung & Cheng 2014	0	0	0	0	0	2		
Yamada et al. 2015	0	0	0	1	1	0	42	
Masayuki et al. 2016	0	0	1	1	5	0	0	106

Supplementary Figure S2. The number of common DEGs from the microarray datasets

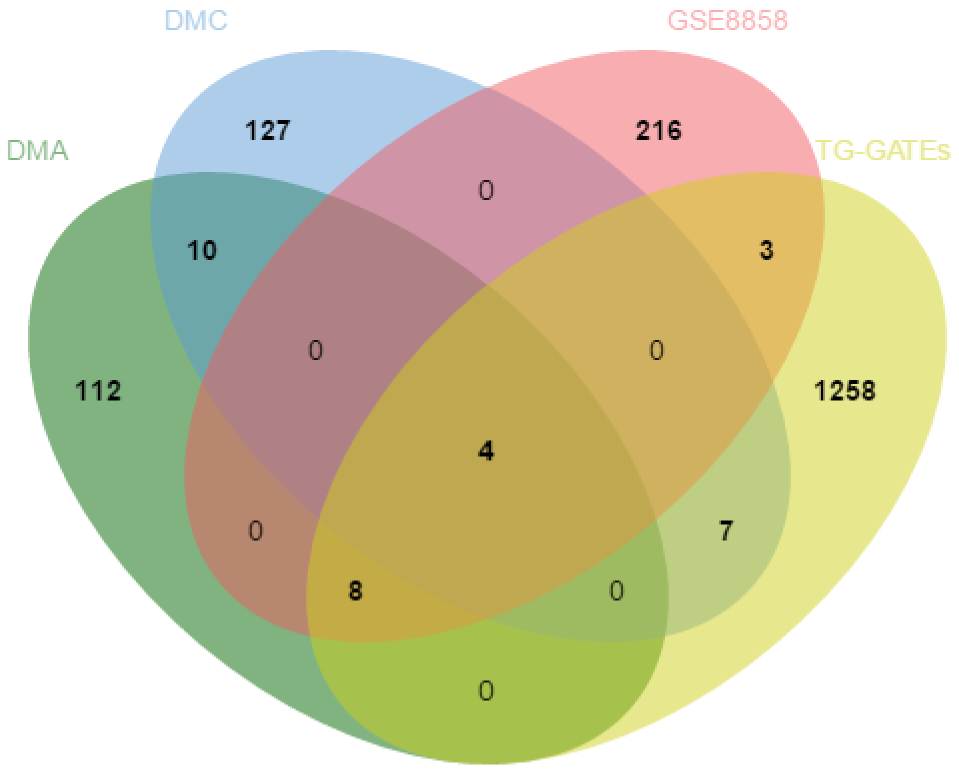

Supplementary Datasets

Supplementary Table S1. Chemical without inconsistent classification

Supplementary Table S2. The average values of consensus biomarkers for four microarray datasets

Supplementary Table S3. The dosage and classification of inconsistently classified chemicals
