# Supplementary material for: Identification of consensus biomarkers for predicting non-genotoxic hepatocarcinogens
Source: Sci Rep. 2017 Jan 24;7:41176. doi: 10.1038/srep41176 (PMC5259716; doi:10.1038/srep41176)
Supplement: Supplementary Information [file srep41176-s2.pdf]

Supplementary Table S1. Chemical without inconsistent classification

Chemical	Nie et al. 2006	Fielden et al. 2007
17-Methyltestosterone		NGHC
1-Amino-2,4-dibromoanthraquinone		
2,3,7,8-Tetrachlorodibenzo-P-Dioxin		NGHC
Acetamide		
Anastrozole		NGHC
Beta-Naphthoflavone		NGHC
Bezafibrate		NGHC
Bis(2-Ethylhexyl)Phthalate		NGHC
Bupropion		NGHC
Carbimazole		NGHC
Carbon Tetrachloride		NGHC
Chloroform		NGHC
Clofibrate		NGHC
Coumarin		
Dehydroepiandrosterone		
Dipyrone		
Estriol		NGHC
Ethinylestradiol	NGHC	NGHC
Ethionine		
Ethisterone		NGHC
Ethylestrenol		NGHC
Fenbendazole		NGHC
Fenofibrate		NGHC
Fluconazole		NGHC
Gemfibrozil		NGHC
Hexachlorobenzene		
Lovastatin		NGHC
Methapyrilene	NGHC	NGHC
Methylcarbamate		
Mifepristone		NGHC
Monocrotaline	NGHC	
Nafenopin		NGHC
Norethindrone		NGHC
Norethindrone Acetate		NGHC
N-vinylpyrrolidone		
Oxfendazole		NGHC
Oxymetholone		NGHC
Pentobarbital		NGHC
Phenobarbital		NGHC
Piperonylbutoxide	NGHC	
Pirinixic Acid		NGHC
Pravastatin		NGHC
Prednisolone		NGHC
Progesterone	NGHC	NGHC
Rimonabant		
Safrole		NGHC

Spironolactone		NGHC
Stanozolol		NGHC
Testosterone		NGHC
Thioacetamide		NGHC
1,1-Dichloroethene		
3-methylcholanthrene		
6-Mercaptopurine		
Acarbose		
Acetazolamide		
Acyclovir		NHC
Adapin		
Ajmaline		
Alfacalcidol		NHC
Allopurinol		
Allyl Alcohol		NHC
Alpha-methyldopa		
Amiodarone hydrochloride	NHC	
Amitriptyline		
Amlodipine		NHC
Amoxapine		NHC
Aniline	NHC	
Ascorbic acid		
Aspirin	NHC	NHC
Atenolol	NHC	
Atorvastatin		NHC
Azathioprine		NHC
Azithromycin		NHC
Bendazac		
Benzbromarone		
Benzethonium Chloride		NHC
Benziodarone		
Benzoic Acid		NHC
Benzothiazyl disulfide		
Beta-hydroxypropyl-cyclodextrin	NHC	
Bisphenol A		NHC
Bithionol		
Bromobenzene		
Bromocryptine	NHC	
Bromoethanamine		
Bucetin		
Buspirone	NHC	
Busulfan		
Butylated hydroxytoluene	NHC	
Caffeine		
Capsaicin		
Captopril	NHC	
Carboplatin		
Carvedilol		NHC
Catechol		NHC

Cefuroxime		
Celecoxib		NHC
Cephalothin		
Cerivastatin		NHC
Chlorambucil		
Chloramphenicol		
Chlormadinone		
Chlormezanone		
Chlorpheniramine		
Chlorpromazine		
Chlorpropamide		
Cholecalciferol		NHC
Choline Chloride		NHC
Cimetidine		
Ciprofloxacin		NHC
Cisplatin		
Citalopram		NHC
Citric Acid		NHC
Clarithromycin		NHC
Clomiphene		NHC
Clomipramine		NHC
Clotrimazole		NHC
Clozapine	NHC	
Colchicine		
Cortisone		NHC
Cycloheximide		NHC
Cyclophosphamide		
Cyclosporin A		NHC
Cytarabine		NHC
Danazol		
Dantrolene	NHC	
Dapsone	NHC	
Dexamethasone	NHC	
Dichlorvos		NHC
Diclofenac		NHC
Dieldrin	NHC	
Diltiazem		
Dipyridamole	NHC	
Disopyramide		
Disulfiram	NHC	
Doxorubicin		
Enalapril	NHC	
Ergocalciferol		NHC
Erythromycin	NHC	NHC
Ethambutol		
Ethylene Glycol		NHC
Etodolac		NHC
Etoposide		NHC
Eugenol		

Famciclovir		NHC
Famotidine	NHC	
Finasteride		NHC
Fluphenazine		
Fluoxetine	NHC	NHC
Flutamide	NHC	
Furosemide	NHC	
Fluvastatin		NHC
Gentamicin		
Gentian Violet		NHC
Geraniol		NHC
Glibenclamide	NHC	
Glimepiride		NHC
Glipizide		NHC
Hexachlorophene		NHC
Hydrazine		
Hydrocortisone		NHC
Hydroxyzine		
Iansoprazole	NHC	
Ibuprofen	NHC	NHC
Ifosfamide		
Imipramine		
Indomethacin	NHC	NHC
Iproniazid		
Isoeugenol		
Isoprenaline		NHC
Isotretinoin		NHC
Itraconazole	NHC	NHC
Ketoconazole	NHC	NHC
Ketorolac		NHC
L-tryptophan		
Labetalol		
Latrepirdine		
Lead (ii) Acetate		
Lead (iv) Acetate		NHC
Levamisole		NHC
Lorazepam		NHC
Lornoxicam		
Mebendazole	NHC	
Mefenamic acid		
Megestrol Acetate		NHC
Meloxicam		NHC
Mestranol		
Metformin	NHC	
Methimazole		
Methotrexate		
Methyldopa	NHC	NHC
Methyltestosterone		
Metoprolol	NHC	

Mexiletine		
Mitomycin c		
Moxisylyte		
Mycophenolic acid	NHC	
Naltrexone	NHC	
Naphthyl isothiocyanate		
Naproxen		
Nevirapine		NHC
Niacin	NHC	
Niacinamide		
Nicotinic acid		
Nifedipine	NHC	
Nimesulide		
Nisoldipine		NHC
Nitrofurantoin	NHC	
Nitrofurazone		
Nizatidine	NHC	
N,N'-Diphenyl-p-phenylenediamine		
Olanzapine		NHC
Omeprazole		NHC
Oxyquinoline		NHC
Papaverine		
Pemoline		NHC
Penicillamine		
Pergolide		NHC
Perhexiline	NHC	NHC
Phenacetin		NHC
Phenothiazine		NHC
Phenylanthranilic acid		
Phenylbutazone		
Phenylephrine	NHC	
Pioglitazone		NHC
Praziquantel		NHC
Primidone		NHC
Procarbazine		
Promethazine		NHC
Propylene Glycol		NHC
Propylthiouracil		NHC
Puromycin aminonucleoside		
Pyrazinamide		NHC
Quercetin	NHC	
Quetiapine		NHC
Quinidine		
Rabeprazole		NHC
Raloxifene	NHC	NHC
Ranitidine	NHC	
Rifabutin		NHC
Rifampin	NHC	
Rofecoxib		NHC

Rosiglitazone	NHC	NHC
Rotenone	NHC	
Roxithromycin		NHC
Sildenafil		NHC
Sparfloxacin		NHC
Streptozotocin		
Sulfamethoxazole	NHC	
Sulindac		
Sulpiride		
Tacrine		
Terbinafine		
Tetracycline	NHC	NHC
Thioridazine		
Theophylline		
Tiopronin		
Ticlopidine		NHC
Tocainide		NHC
Tolazamide		NHC
Tolbutamide		
Tretinoin		NHC
Triazolam		
Trichloroacetic Acid		NHC
Trimethadione		
Troglitazone	NHC	NHC
Valproic Acid	NHC	NHC
Vancomycin		
Venlafaxine		NHC
Verapamil	NHC	
Vinblastine		NHC
Vinorelbine		NHC
Vitamin A	NHC	
Zidovudine		NHC

References:

Nie, A. Y. et al. Predictive toxicogenomics approaches reveal underlying molecu

Fielden, M. R., Brennan, R. & Gollub, J. A gene expression biomarker provides

Nioi, P., Pardo, I. D. R., Sherratt, P. J. & Snyder, R. D. Prediction of non-genoto

Uehara, T. et al. A toxicogenomics approach for early assessment of potential no

Auerbach, S. S. et al. Predicting the hepatocarcinogenic potential of alkenylbenz

Fielden, M. R. et al. Development and evaluation of a genomic signature for the

Uehara, T. et al. Prediction model of potential hepatocarcinogenicity of rat hepa

Liu, Z., Kelly, R., Fang, H., Ding, D. & Tong, W. Comparative analysis of predi

Yamada, F. et al. Toxicogenomics discrimination of potential hepatocarcinogeni

Romer, M. *et al.* Cross-platform toxicogenomics for the prediction of non-gen

	Database		
Nioi et al. 2008	Uehara et al. 2008	Auerbach et al. 2010	Fielden et al. 2011
		NGHC	
			NGHC
			NGHC
			NGHC
			NGHC
			NGHC
			NGHC
	NGHC		
			NGHC
	NGHC		
	NGHC		
	NGHC		
			NGHC
			NGHC
			NGHC
	NGHC		
	NGHC		
NGHC	NGHC		NGHC
			NGHC
			NGHC
			NGHC
			NGHC
NGHC	NGHC		NGHC
			NGHC
			NGHC

NGHC

NGHC

NHC

NHC

NHC

NHC

NHC

NHC

NHC

NHC

NHC

NHC

NHC

NHC

NHC

NHC

NHC

NHC

NHC

NHC

NHC
NHC
NHC
NHC
NHC

NHC
NHC

NHC
NHC NHC

NHC
NHC

NHC

NHC

NHC

NHC NHC

NHC

NHC

NHC

NHC

NHC

NHC

NHC

NHC

NHC
NHC

NHC

NHC

NHC
NHC
NHC

NHC
NHC
NHC NHC

NHC

NHC

NHC

NHC

NHC

NHC

NHC

NHC

NHC

NHC

NHC

NHC NHC

---

ular mechanisms of nongenotoxic carcinogenicity. Molecular carcinogenesis 45, 914-93
early prediction and mechanistic assessment of hepatic tumor induction by nongenotox
oxic carcinogenesis in rats using changes in gene expression following acute dosing. *Ch*
on-genotoxic hepatocarcinogenicity of chemicals in rats. Toxicology 250, 15-26, doi:10.
zene flavoring agents using toxicogenomics and machine learning. Toxicology and appli
prediction and mechanistic assessment of nongenotoxic hepatocarcinogens in the rat. T
tocarcinogens using a large-scale toxicogenomics database. Toxicology and applied pha
ctive models for nongenotoxic hepatocarcinogenicity using both toxicogenomics and qu
icity of non-genotoxic compounds in rat liver. Journal of applied toxicology : JAT 33, 1
otoxic hepatocarcinogenesis in rat. *PloS one* **9**, e97640, doi:10.1371/journal.pone.009

Uehara et al. 2011	Liu et al. 2011	Yamada et al. 2013	Römer et al. 2014
			NGHC
	NGHC		
NGHC	NGHC	NGHC	
	NGHC		
NGHC		NGHC	
NGHC		NGHC	
			NGHC
	NGHC		
NGHC		NGHC	
NGHC		NGHC	NGHC
NGHC		NGHC	
NGHC		NGHC	
NGHC		NGHC	
NGHC		NGHC	NGHC
			NGHC
NGHC		NGHC	
	NGHC		
NGHC	NGHC	NGHC	NGHC
			NGHC
	NGHC		
	NGHC		

	NHC	
	NHC	
	NHC	
NHC		
NHC		
NHC		
NHC		
NHC		
NHC	NHC	NHC
		NHC
NHC		
NHC		
NHC	NHC	NHC
NHC	NHC	
NHC		
NHC		
NHC		
	NHC	
	NHC	
	NHC	
NHC		
NHC		
NHC		NHC
	NHC	
NHC		
	NHC	
NHC		NHC
NHC		
	NHC	

			NHC
NHC			
	NHC		
NHC			
NHC			
NHC			
NHC		NHC	
NHC			
NHC		NHC	
	NHC		
NHC			
NHC		NHC	
NHC			
NHC			
NHC			
	NHC		
NHC			
NHC	NHC	NHC	
NHC			
NHC			
	NHC		
NHC			
NHC		NHC	
NHC			
NHC			
NHC			
NHC		NHC	
	NHC		
NHC			
NHC			
	NHC		
NHC			

NHC		NHC
NHC		
NHC		
NHC		
NHC		
NHC		
	NHC	
	NHC	
	NHC	
NHC		
NHC	NHC	NHC
	NHC	
NHC		
NHC		NHC
NHC		NHC
NHC		NHC
NHC		NHC
	NHC	
	NHC	
NHC		NHC
NHC		
NHC		NHC
	NHC	
NHC		NHC
NHC		
NHC	NHC	NHC
NHC		
NHC		

NHC		NHC	
	NHC		
NHC			
NHC			
NHC			
	NHC		
	NHC		
NHC		NHC	
NHC		NHC	NHC
NHC			
NHC	NHC		
NHC			
	NHC		
NHC	NHC		
	NHC		
NHC			
NHC			
NHC			
NHC			
NHC	NHC		
	NHC		
NHC			
NHC			
	NHC		
	NHC		
NHC			
	NHC		
NHC	NHC		
NHC			
	NHC		
NHC			
NHC		NHC	

	NHC	
NHC		NHC
NHC		
NHC		
NHC		
NHC		NHC
NHC		
NHC		NHC
NHC		
NHC		
	NHC	
NHC		
	NHC	
NHC		
NHC		NHC
NHC		
NHC		
	NHC	
NHC		NHC

3, doi:10.1002/mc.20205 (2006).
ic chemicals. Toxicological sciences : an official journal of the Society of Toxicology 99, 90-100,
em-Biol Interact **172**, 206-215, doi:DOI 10.1016/j.cbi.2008.01.009 (2008).
.1016/j.tox.2008.05.013 (2008).
ed pharmacology 243, 300-314, doi:DOI 10.1016/j.taap.2009.11.021 (2010).
oxicological sciences : an official journal of the Society of Toxicology 124, 54-74, doi:10.1093/to
rmacology 255, 297-306, doi:10.1016/j.taap.2011.07.001 (2011).
antitative structure-activity relationships. Chemical research in toxicology 24, 1062-1070, doi:10.
284-1293, doi:10.1002/jat.2790 (2013).
7640 (2014).

doi:10.1093/toxsci/kfm156 (2007).

xsci/kfr202 (2011).

1021/tx2000637 (2011).
