# Supplementary material for: Identification of consensus biomarkers for predicting non-genotoxic hepatocarcinogens
Source: Sci Rep. 2017 Jan 24;7:41176. doi: 10.1038/srep41176 (PMC5259716; doi:10.1038/srep41176)
Supplement: Supplementary Information [file srep41176-s3.pdf]

Supplementary Table S2. The average values of consensus biomarkers for four microarray data

Gene	DMA		DMC		GSE8858		TG-GATEs	
	NGHCs	NHCs	NGHCs	NHCs	NGHCs	NHCs	NGHCs	NHCs
A2m	8.07	9.5	8.7	9.84	8.67	9.77	7.11	8.08
Ca3	11.59	12.18	13.31	14.27	13.32	14.4	10.75	11.62
Cxcl1	6.45	7.53	7.74	8.7	7.73	8.64	5.65	6.45
Cyp8b1	10.52	11.5	7.33	8.14	7.21	8.14	10.28	10.89

The expression values were log2-transformed.
