# Supplementary material for: Identification of consensus biomarkers for predicting non-genotoxic hepatocarcinogens
Source: Sci Rep. 2017 Jan 24;7:41176. doi: 10.1038/srep41176 (PMC5259716; doi:10.1038/srep41176)
Supplement: Supplementary Information [file srep41176-s4.pdf]

Supplementary Table S3. The dosage and classification of inconsistently classified chemicals

Chemical	DrugMatrixCodelink	DrugMatrixAffymetrix
17-methyltestosterone	2000 mg/kg	
2,3,7,8-tetrachlorodibenzo-p-dioxin	0.02 mg/kg	0.02 mg/kg
Anastrozole	400 mg/kg	400 mg/kg
Beta-naphthoflavone	1500 mg/kg	1500 mg/kg
Bezafibrate	617 mg/kg	617 mg/kg
Bis(2-ethylhexyl)phthalate	1000 mg/kg	
Bupropion	895 mg/kg	
Carbimazole	400 mg/kg	400 mg/kg
Carbon tetrachloride	3178 mg/kg	1175 mg/kg
Chloroform	600 mg/kg	600 mg/kg
Clofibrate	500 mg/kg	
Coumarin		
Dipyrone	1636 mg/kg	
Estriol	313 mg/kg	313 mg/kg
Ethinylestradiol	1480 mg/kg	1480 mg/kg
Ethionine		
Ethisterone	1500 mg/kg	1500 mg/kg
Ethylestrenol	390 mg/kg	
Fenbendazole	375 mg/kg	375 mg/kg
Fenofibrate	215 mg/kg	
Fluconazole	394 mg/kg	394 mg/kg
Gemfibrozil	700 mg/kg	700 mg/kg
Hexochlorobenzene		
Lovastatin	1500 mg/kg	1500 mg/kg
Methapyrilene	100 mg/kg	
Mifepristone	300 mg/kg	300 mg/kg
Monocrotaline		
Nafenopin	338 mg/kg	338 mg/kg
Norethindrone	375 mg/kg	
Norethindrone acetate	125 mg/kg	125 mg/kg
Oxfendazole	1500 mg/kg	1500 mg/kg
Oxymetholone	1170 mg/kg	1170 mg/kg
Pentobarbital	70 mg/kg	
Phenobarbital	80 mg/kg	54 mg/kg
Pirinixic acid	364 mg/kg	364 mg/kg
Pravastatin	1200 mg/kg	
Prednisolone	184 mg/kg	184 mg/kg
Progesterone	164 mg/kg	164 mg/kg
Safrole	488 mg/kg	488 mg/kg
Spironolactone	300 mg/kg	300 mg/kg
Stanozolol	150 mg/kg	
Testosterone	375 mg/kg	
Thioacetamide	200 mg/kg	
1,1-dichloroethene	600 mg/kg	600 mg/kg
3-methylcholanthrene	300 mg/kg	300 mg/kg
6-mercaptopurine	25 mg/kg	
Acarbose		

Acetazolamide	250 mg/kg	
Acyclovir	980 mg/kg	
Ajmaline		
Alfacalcidol	0.043 mg/kg	
Allopurinol		
Allyl alcohol	32 mg/kg	32 mg/kg
Amiodarone	147 mg/kg	
Amitriptyline		
Amlodipine	19 mg/kg	
Amoxapine	313 mg/kg	
Aspirin	500 mg/kg	
Atorvastatin	300 mg/kg	2.5 mg/kg
Azathioprine	160 mg/kg	
Azithromycin	225 mg/kg	
Bendazac		
Benzbromarone		
Benzethonium chloride	30 mg/kg	
Benziodarone		
Benzoic acid	1700 mg/kg	
Benzothiazyl disulfide	2600 mg/kg	2600 mg/kg
Bisphenol A	610 mg/kg	610 mg/kg
Bithionol	333 mg/kg	333 mg/kg
Bromobenzene		
Bucetin		
Busulfan	36 mg/kg	
Caffeine		
Capsaicin	35 mg/kg	
Captopril		
Carboplatin	14 mg/kg	
Carvedilol	2000 mg/kg	2000 mg/kg
Catechol	195 mg/kg	195 mg/kg
Celecoxib	400 mg/kg	
Cephalothin		
Cerivastatin	7 mg/kg	7 mg/kg
Chlorambucil	4.5 mg/kg	
Chloramphenicol		
Chlormadinone		
Chlormezanone		
Chlorpheniramine		
Chlorpromazine	73 mg/kg	
Chlorpropamide		
Cholecalciferol	8 mg/kg	
Choline chloride	2550 mg/kg	
Cimetidine		
Ciprofloxacin	450 mg/kg	
Cisplatin	2 mg/kg	
Citalopram	90 mg/kg	
Citric acid	3000 mg/kg	
Clarithromycin	476 mg/kg	

Clomiphene	250 mg/kg	
Clomipramine	115 mg/kg	
Clotrimazole	178 mg/kg	
Cortisone	206 mg/kg	206 mg/kg
Cycloheximide	0.25 mg/kg	
Cyclophosphamide		
Cyclosporine A	350 mg/kg	
Cytarabine	487 mg/kg	
Danazol	2000 mg/kg	
Dantrolene		
Dexamethasone	150 mg/kg	150 mg/kg
Dichlorvos	17 mg/kg	
Diclofenac	10 mg/kg	10 mg/kg
Diltiazem		
Dipyridamole	750 mg/kg	
Disopyramide		
Disulfiram	500 mg/kg	
Doxorubicin	3 mg/kg	3 mg/kg
Enalapril		
Ergocalciferol	15 mg/kg	
Erythromycin	1500 mg/kg	1500 mg/kg
Ethambutol		
Ethylene glycol	3525 mg/kg	
Etodolac	24 mg/kg	24 mg/kg
Etoposide	188 mg/kg	
Famciclovir	1200 mg/kg	
Famotidine		
Finasteride	800 mg/kg	
Fluoxetine	52 mg/kg	
Fluphenazine	22 mg/kg	22 mg/kg
Flutamide		
Fluvastatin	94 mg/kg	94 mg/kg
Gentamicin	267 mg/kg	
Gentian violet	18 mg/kg	18 mg/kg
Geraniol	1500 mg/kg	
Glibenclamide		
Glimepiride	2500 mg/kg	
Glipizide	2500 mg/kg	
Hexachlorophene	8 mg/kg	8 mg/kg
Hydrazine	45 mg/kg	
Hydrocortisone	56 mg/kg	56 mg/kg
Hydroxyzine		
Ibuprofen	263 mg/kg	263 mg/kg
Ifosfamide	143 mg/kg	143 mg/kg
Imipramine		
Indomethacin	12 mg/kg	
Iproniazid		
Isoeugenol	1560 mg/kg	1560 mg/kg
Isoprenaline	15 mg/kg	

Isotretinoin	125 mg/kg	
Itraconazole	1093 mg/kg	1093 mg/kg
Ketoconazole	227 mg/kg	227 mg/kg
Ketorolac	48 mg/kg	48 mg/kg
Labetalol		
Lead(ii)acetate	600 mg/kg	
Lead(iv)acetate	600 mg/kg	
Levamisole	120 mg/kg	
Lorazepam	2000 mg/kg	2000 mg/kg
Mebendazole	714 mg/kg	714 mg/kg
Mefenamic acid	93 mg/kg	
Megestrol acetate	132 mg/kg	132 mg/kg
Meloxicam	33 mg/kg	33 mg/kg
Mestranol	250 mg/kg	250 mg/kg
Metformin		
Methimazole	100 mg/kg	100 mg/kg
Methotrexate	27 mg/kg	0.3 mg/kg
Methyldopa	325 mg/kg	
Methyltestosterone		
Mexiletine		
Mitomycin C	1.7 mg/kg	1.7 mg/kg
Moxisylyte		
N,N'-diphenyl-p-phenylenediamine	1000 mg/kg	
Naphthyl_isothiocyanate		
Naproxen	134 mg/kg	
Nevirapine	200 mg/kg	
Niacin	2625 mg/kg	
Niacinamide	750 mg/kg	
Nicotinic acid		
Nifedipine		
Nimesulide	162 mg/kg	
Nisoldipine	1125 mg/kg	
Nitrofurantoin	76 mg/kg	
Nitrofurazone		
Olanzapine	23 mg/kg	
Omeprazole	415 mg/kg	
Oxyquinoline	68 mg/kg	
Papaverine		
Pemoline	70 mg/kg	70 mg/kg
Penicillamine	852 mg/kg	
Pergolide	1.1 mg/kg	
Perhexiline	320 mg/kg	
Phenacetin	619 mg/kg	
Phenothiazine	386 mg/kg	386 mg/kg
Phenylanthranilic acid		
Phenylbutazone		
Pioglitazone	1500 mg/kg	
Praziquantel	1200 mg/kg	
Primidone	750 mg/kg	750 mg/kg

Procarbazine	54 mg/kg	54 mg/kg
Promethazine	113 mg/kg	
Propylene glycol	2000 mg/kg	
Propylthiouracil	625 mg/kg	625 mg/kg
Pyrazinamide	1500 mg/kg	
Quetiapine	500 mg/kg	
Quinidine		
Rabeprazole	1024 mg/kg	
Raloxifene	650 mg/kg	650 mg/kg
Ranitidine		
Rifabutin	1500 mg/kg	
Rofecoxib	1550 mg/kg	
Rosiglitazone	1800 mg/kg	
Rotenone		
Roxithromycin	312 mg/kg	312 mg/kg
Sildenafil	420 mg/kg	
Sparfloxacin	450 mg/kg	
Streptozotocin	138 mg/kg	138 mg/kg
Sulindac	132 mg/kg	132 mg/kg
Sulpiride		
Tacrine	24 mg/kg	
Terbinafine	2000 mg/kg	
Tetracycline	1500 mg/kg	
Theophylline		
Thioridazine		
Ticlopidine	223 mg/kg	
Tocainide	224 mg/kg	
Tolazamide	1500 mg/kg	
Tolbutamide		
Tretinoin	7 mg/kg	
Triazolam		
Trichloroacetic acid	474 mg/kg	474 mg/kg
Trimethadione		
Troglitazone	1200 mg/kg	
Venlafaxine	320 mg/kg	
Vinblastine	0.3 mg/kg	0.3 mg/kg
Vinorelbine	1.5 mg/kg	1.5 mg/kg
Zidovudine	1540 mg/kg	

GSE8858	TG-GATEs	Class
2000mg/kg		NGHC
0.02 mg/kg		NGHC
400 mg/kg		NGHC
1500mg/kg		NGHC
617mg/kg		NGHC
1000 mg/kg		NGHC
895 mg/kg		NGHC
400 mg/kg		NGHC
	300 mg/kg	NGHC
600 mg/kg		NGHC
500 mg/kg	300 mg/kg	NGHC
	150 mg/kg	NGHC
1636mg/kg		NGHC
313 mg/kg		NGHC
1480 mg/kg	10 mg/kg	NGHC
	250 mg/kg	NGHC
1500 mg/kg		NGHC
390 mg/kg		NGHC
375 mg/kg		NGHC
	1000 mg/kg	NGHC
394 mg/kg		NGHC
700 mg/kg	300 mg/kg	NGHC
	2000 mg/kg	NGHC
1500 mg/kg		NGHC
100 mg/kg	100 mg/kg	NGHC
300 mg/kg		NGHC
	30 mg/kg	NGHC
338 mg/kg		NGHC
375 mg/kg		NGHC
		NGHC
1500 mg/kg		NGHC
1170 mg/kg		NGHC
70 mg/kg		NGHC
80 mg/kg	300 mg/kg	NGHC
		NGHC
1200 mg/kg		NGHC
184 mg/kg		NGHC
164 mg/kg		NGHC
488 mg/kg		NGHC
300 mg/kg		NGHC
150 mg/kg		NGHC
375 mg/kg		NGHC
200 mg/kg	45 mg/kg	NGHC
600 mg/kg		NHC
300 mg/kg		NHC
25 mg/kg		NHC
	1000 mg/kg	NHC

250 mg/kg	600 mg/kg	NHC
980 mg/kg		NHC
	300 mg/kg	NHC
0.043 mg/kg		NHC
	150 mg/kg	NHC
	30 mg/kg	NHC
147 mg/kg	2000 mg/kg	NHC
	150 mg/kg	NHC
19 mg/kg		NHC
313 mg/kg		NHC
500 mg/kg	2000 mg/kg	NHC
300 mg/kg		NHC
160 mg/kg	30 mg/kg	NHC
225 mg/kg		NHC
	1000 mg/kg	NHC
	200 mg/kg	NHC
		NHC
	300 mg/kg	NHC
		NHC
		NHC
		NHC
333 mg/kg		NHC
	300 mg/kg	NHC
	2000 mg/kg	NHC
36 mg/kg		NHC
	100 mg/kg	NHC
35 mg/kg		NHC
	1000 mg/kg	NHC
14 mg/kg	100 mg/kg	NHC
2000 mg/kg		NHC
195 mg/kg		NHC
400 mg/kg		NHC
	2000 mg/kg	NHC
7 mg/kg		NHC
4.5 mg/kg		NHC
	1000 mg/kg	NHC
	2000 mg/kg	NHC
	500 mg/kg	NHC
	30 mg/kg	NHC
73 mg/kg		NHC
	300 mg/kg	NHC
8 mg/kg		NHC
		NHC
	1000 mg/kg	NHC
450 mg/kg	1000 mg/kg	NHC
2 mg/kg	3 mg/kg	NHC
90 mg/kg		NHC
		NHC
476 mg/kg		NHC

250 mg/kg		NHC
115 mg/kg	100 mg/kg	NHC
178 mg/kg		NHC
206 mg/kg		NHC
0.25 mg/kg		NHC
	150 mg/kg	NHC
	300 mg/kg	NHC
487 mg/kg		NHC
2000 mg/kg	2000 mg/kg	NHC
	250 mg/kg	NHC
150 mg/kg		NHC
17 mg/kg		NHC
10 mg/kg	100 mg/kg	NHC
	800 mg/kg	NHC
750 mg/kg		NHC
	400 mg/kg	NHC
500 mg/kg	600 mg/kg	NHC
3 mg/kg	10 mg/kg	NHC
	600 mg/kg	NHC
15 mg/kg		NHC
1500 mg/kg	1000 mg/kg	NHC
	1000 mg/kg	NHC
		NHC
24 mg/kg		NHC
188 mg/kg	1000 mg/kg	NHC
1200 mg/kg		NHC
	1000 mg/kg	NHC
800 mg/kg		NHC
52 mg/kg	30 mg/kg	NHC
22 mg/kg	20 mg/kg	NHC
	150 mg/kg	NHC
94 mg/kg		NHC
267 mg/kg	100 mg/kg	NHC
		NHC
1500 mg/kg		NHC
	1000 mg/kg	NHC
2500 mg/kg		NHC
2500 mg/kg		NHC
8 mg/kg		NHC
45 mg/kg		NHC
56 mg/kg		NHC
	100 mg/kg	NHC
275mg/kg	400 mg/kg	NHC
143 mg/kg		NHC
	100 mg/kg	NHC
12 mg/kg	50 mg/kg	NHC
	60 mg/kg	NHC
1560 mg/kg		NHC
15 mg/kg		NHC

125 mg/kg		NHC
1093 mg/kg		NHC
227 mg/kg	100 mg/kg	NHC
48 mg/kg		NHC
	450 mg/kg	NHC
		NHC
		NHC
120 mg/kg		NHC
2000 mg/kg		NHC
714 mg/kg		NHC
	300 mg/kg	NHC
		NHC
33 mg/kg	100 mg/kg	NHC
250 mg/kg		NHC
	1000 mg/kg	NHC
100 mg/kg	100 mg/kg	NHC
27 mg/kg		NHC
325 mg/kg	600 mg/kg	NHC
	300 mg/kg	NHC
	400 mg/kg	NHC
		NHC
	500 mg/kg	NHC
1000 mg/kg		NHC
	150 mg/kg	NHC
134 mg/kg	200 mg/kg	NHC
200 mg/kg		NHC
2625 mg/kg		NHC
750 mg/kg		NHC
	1000 mg/kg	NHC
	1000 mg/kg	NHC
162 mg/kg	300 mg/kg	NHC
1125 mg/kg		NHC
76 mg/kg	600 mg/kg	NHC
	300 mg/kg	NHC
23 mg/kg		NHC
415 mg/kg	1000 mg/kg	NHC
68 mg/kg		NHC
	400 mg/kg	NHC
70 mg/kg	75 mg/kg	NHC
852 mg/kg	1000 mg/kg	NHC
1.1 mg/kg		NHC
320 mg/kg	150 mg/kg	NHC
619 mg/kg	2000 mg/kg	NHC
386 mg/kg		NHC
	2000 mg/kg	NHC
	200 mg/kg	NHC
1500 mg/kg		NHC
1200 mg/kg		NHC
750 mg/kg		NHC

54 mg/kg		NHC
113 mg/kg	200 mg/kg	NHC
		NHC
625 mg/kg	100 mg/kg	NHC
1500 mg/kg		NHC
500 mg/kg		NHC
	200 mg/kg	NHC
1024 mg/kg		NHC
650 mg/kg		NHC
	1000 mg/kg	NHC
1500 mg/kg		NHC
1550 mg/kg		NHC
1800 mg/kg	500 mg/kg	NHC
	50 mg/kg	NHC
312 mg/kg		NHC
420 mg/kg		NHC
450 mg/kg		NHC
138 mg/kg		NHC
132 mg/kg	150 mg/kg	NHC
	2000 mg/kg	NHC
24 mg/kg	30 mg/kg	NHC
2000 mg/kg	750 mg/kg	NHC
1500 mg/kg	1000 mg/kg	NHC
	200 mg/kg	NHC
	100 mg/kg	NHC
223 mg/kg	1000 mg/kg	NHC
224 mg/kg		NHC
1500 mg/kg		NHC
	1000 mg/kg	NHC
7 mg/kg		NHC
	1000 mg/kg	NHC
		NHC
	500 mg/kg	NHC
1200 mg/kg		NHC
320 mg/kg		NHC
0.3 mg/kg		NHC
1.5 mg/kg		NHC
1540 mg/kg		NHC
